# The Relationship Between Pain and Depression in Fibromyalgia: Structural Equation Modeling and Network Analysis

**DOI:** 10.3390/ijerph23030316

**Published:** 2026-03-04

**Authors:** Francesco Oliva, Mariagrazia Merola, Alberto Olivero, Paolo Leombruni

**Affiliations:** 1 Department of Clinical and Biological Sciences, University of Turin, Regione Gonzole 10, 10043 Orbassano, Italy; francesco.oliva@unito.it (F.O.); mariagrazia.merola@unito.it (M.M.); 2 Department of Neurosciences “Rita Levi Montalcini”, University of Turin, Via Cherasco 15, 10126 Torino, Italy; paolo.leombruni@unito.it

**Keywords:** fibromyalgia, depression, chronic pain, fatigue, psychopathology

## Abstract

**Highlights:**

**Public health relevance—How does this work relate to a public health issue?**

- Fibromyalgia contributes to disability, productivity loss, and healthcare utilization within the broader public health burden of chronic pain, particularly when comorbid depressive symptoms are present.
- This study investigates how depression, fatigue, stress, and core fibromyalgia domains structurally interact, providing insight into symptom configurations linked to functional impairment and service needs.

**Public health significance—Why is this work of significance to public health?**

- By integrating structural equation modeling and network analysis, this study identifies depressive symptoms as a structurally prominent dimension bridging physical and psychological components of fibromyalgia.
- Clarifying the role of depression and fatigue within the fibromyalgia symptom architecture helps explain why comorbid depression is consistently associated with disability and increased healthcare demands in chronic pain populations.

**Public health implications—What are the key implications or messages for practitioners, policy makers and/or researchers in public health?**

- Systematic identification of depressive symptoms within fibromyalgia and chronic pain services may represent a clinically and organizationally relevant strategy for addressing high functional burden.
- Integrated and multidisciplinary care pathways that incorporate mental health assessment alongside pain and fatigue management are consistent with the observed symptom structure and may inform health-service planning in chronic pain management.

**Abstract:**

Fibromyalgia (FM) is characterized by chronic pain, fatigue, and sleep disturbances, which often coexist with psychiatric symptoms, complicating its clinical profile. This study aims to investigate the relationships between FM components and psychopathological correlates, focusing on the central role of depression within the FM symptom network. A cross-sectional study was conducted on 50 outpatients diagnosed with FM according to the American College of Rheumatology 2016 criteria. Participants completed a comprehensive battery of validated assessment tools measuring FM components (pain, fatigue, and sleep disturbances), psychopathology (depression and anxiety), and stress-related dimensions. Structural equation modeling (SEM) and network analysis were used to explore the interplay between FM-related and psychological factors. The findings revealed a complex relationship between depression and pain in FM, with depression emerging as a central and highly connected factor within the symptom network, associated with both emotional and physical dysfunction. Fatigue was identified as a significant mediator between depression and pain, while stress, though not central, contributed to the overall symptom burden. These interactions underscore the multifaceted nature of FM, where psychological and physical symptoms are intricately interconnected through shared mechanisms. Systematic assessment of depressive symptoms may represent a clinically relevant target and a potential leverage point for integrated care pathways in chronic pain services.

## 1. Introduction

Fibromyalgia (FM) is a multifaceted syndrome characterized by chronic widespread pain [1] or pain affecting multiple anatomical sites [2]. This condition is accompanied by persistent fatigue, sleep disturbances, and functional impairment in the absence of clear organic causes. FM prevalence varies significantly, ranging from 0.8% to 9.3% across countries and diagnostic criteria, with an estimated global prevalence of approximately 2.7% [2].

Pain in FM may occur anywhere in the body and often presents with mixed sensory characteristics, such as paresthesias and hyperalgesia. Fatigue affects both physical and mental functioning across a wide spectrum of severity, substantially impacting daily life. Sleep disturbances typically include insomnia and nonrestorative sleep, further contributing to overall symptom burden [2,3].

Beyond its clinical presentation, FM represents a relevant public health challenge due to its association with substantial disability, reduced health-related quality of life, and work productivity loss, alongside increased healthcare utilization and considerable direct and indirect societal costs [4,5,6,7]. This burden aligns with epidemiological evidence on chronic pain, including high-impact chronic pain, which contributes markedly to disability and healthcare demand [4,5].

In addition, FM is frequently comorbid and related to other chronic pain syndromes and psychiatric conditions [8,9]. Depression is the most prevalent psychiatric comorbidity, with lifetime prevalence estimates of depressive symptoms reported as high as approximately 90% and major depressive disorder (MDD) affecting 62–86% of FM patients. At any given time, depressive symptoms are present in approximately 40% of individuals with FM. The high prevalence has led some researchers to classify FM within the spectrum of “affective spectrum disorders” [9,10,11]. However, whether depressive symptomatology should be conceptualized primarily as a comorbid condition or as a closely interconnected component of the FM clinical phenotype remains a matter of debate. From a population and health-system perspective, depressive symptoms in chronic pain populations are associated with worse functional outcomes and increased healthcare utilization and costs, thereby amplifying the overall burden beyond symptom severity alone [12,13,14,15]. Although the relationship between depression and FM remains unclear, evidence points to a significant association between pain severity and depressive symptoms [16]. Furthermore, improvements in depressive symptoms often correlate with reductions in pain and other FM outcomes [17]. From a therapeutic standpoint, antidepressant agents are commonly employed in FM management, and recent quantitative syntheses have examined their comparative efficacy and tolerability [18,19]. While such findings support the clinical relevance of mood-targeted interventions, they also highlight heterogeneity in treatment response and underscore the need to better understand how depressive symptoms interact with core FM domains such as pain, fatigue, and sleep disturbance.

In FM patients, depressive symptoms are strongly associated with elevated stress levels and increased pain [20]. Research suggests that perceived stress may mediate the relationship between depression and FM symptoms such as fatigue [21]. Similarly, stress is proposed to bridge anxiety with FM symptoms like pain, fatigue, and sleep disturbances [22].

The role of stress in the onset and persistence of FM symptoms is well-documented [23,24,25,26,27,28], including from a molecular perspective [29,30]. Nevertheless, the structural relationships among pain, depression, stress, and other FM-related dimensions remain insufficiently clarified [31]. Many previous investigations have relied on bivariate associations or single analytical approaches, limiting the ability to disentangle modeled interrelationships from the broader organization of symptoms within the FM phenotype. Structural equation modeling (SEM) allows the testing of theoretically informed models, including potential mediational associations and latent constructs of pain. However, SEM requires specifying an a priori structure. In contrast, network analysis conceptualizes symptoms and clinical dimensions as mutually interacting components within a system and identifies central or bridging nodes without assuming latent variables. Combining these approaches may, therefore, provide complementary insights into the role of depressive symptoms within the multidimensional clinical profile of FM. Clarifying how depressive symptoms relate to core FM domains may also inform integrated care strategies and care pathway planning in chronic pain management, where mental health comorbidity is increasingly recognized as a key driver of disability and service needs [32].

In light of these considerations, the present study aims to investigate the relationship between fibromyalgia components and psychopathological correlates in a sample of outpatients diagnosed with FM, with a particular focus on the role of depression within the broader symptom network.

## 2. Methods

### 2.1. Design and Study Sample

The present cross-sectional study was conducted at the Clinical Psychology Unit of “Città della Salute e della Scienza di Torino” University Hospital (Turin, Italy), between May 2022 and March 2024, on a consecutive sample of patients attending the FM outpatient service.

According to the standard clinical practice of the outpatient unit, patients receiving an FM diagnosis from a rheumatologist were referred to a psychiatrist of the outpatient service for an initial assessment. At the end of the assessment, all the eligible patients received detailed information about the study’s purpose, procedures, risks, and benefits. Those who agreed to participate provided written informed consent and were assigned a unique identification code to ensure anonymity and confidentiality. The study was conducted in compliance with the Declaration of Helsinki, and it was approved by the Research Ethics Committee of the “Città della Salute e della Scienza di Torino” University Hospital, Turin, Italy (protocol #0022712, 27 February 2019).

The study inclusion criteria required participants to be at least 18 years old and to have a diagnosis of FM confirmed by a rheumatology specialist according to the American College of Rheumatology 2016 criteria [1]. Patients with psychiatric conditions requiring urgent clinical intervention, cognitive impairment, or inability to understand and speak the Italian language were excluded.

### 2.2. Assessment Tools

During the enrollment, we collected socio-demographic characteristics (age, gender, education level, employment, living condition and marital status), physiological and lifestyle data (Body Mass Index—BMI, dietary regimen, smoking habit, alcohol and substance use), and anamnestic information about eventual medical and psychiatric comorbid conditions.

Subsequently, we asked the participants to complete a clinical assessment through the following validated assessment tools.

#### 2.2.1. FM Components (Pain, Fatigue, Sleep Quality, and Impact on Daily Life)

The Brief Pain Inventory (BPI) is a nine-item self-administered questionnaire, assessing chronic pain in various conditions [33,34]. It evaluates pain severity (BPI-S) and pain interference in daily activities (BPI-I), with each item rated from 0 (no pain) to 10 (worst pain imaginable). The Italian version of BPI showed good internal consistencies (alpha coefficients < 0.75 for both subscales) and reliability [35].

The Pain Catastrophizing Scale (PCS). This validated self-report scale is used to assess the extent of catastrophizing regarding pain-related thoughts among adults [36]. It encompasses 13 statements reflecting various thoughts and emotions individuals may encounter when confronting pain, covering the three primary dimensions of this concept: rumination (PCS-R), magnification (PCS-M), and helplessness (PCS-H). The cumulative score, obtained by summing the scores of each item rated on a 5-point scale, provides an indication of the overall level of catastrophizing; a total score exceeding 30 signifies a clinically significant level of pain catastrophizing. The Italian validated version [37] exhibits strong internal consistency (α = 0.92) and demonstrates high test–retest reliability (ICC = 0.842).

The Fatigue Assessment Scale (FAS). The FAS is a 10-item self-report scale designed to assess the chronic fatigue scale encompassing both physical and mental fatigue symptoms [38]. Each item on the FAS is rated using a five-point Likert-type scale, ranging from 0 (“never”) to 4 (“always”). Total scores on the FAS provide an indication of the overall severity of fatigue experienced by the individual, ranging from 10 (indicating the lowest level of fatigue) to 50 (denoting the highest level of fatigue). Widely employed in diverse clinical and research settings, the FAS is utilized to evaluate fatigue across various populations, including individuals with chronic illnesses, neurological disorders, and psychiatric conditions. It has demonstrated robust reliability and validity in measuring fatigue severity and assessing treatment outcomes [39].

The Pittsburgh Sleep Quality Index (PSQI) is a self-report assessment tool designed to measure sleep quality and disturbances over a one-month period [40]. It consists of 19 items that generate seven “component” scores: subjective sleep quality, sleep latency, sleep duration, habitual sleep efficiency, sleep disturbances, use of sleeping medication, and daytime dysfunction. Each component is scored on a scale from 0 to 3, with higher scores indicating poorer sleep quality. The sum of the scores for these seven components yields one global score, which ranges from 0 to 21. A total PSQI score greater than 5 has been validated as being highly sensitive and specific in distinguishing good from poor sleepers across a number of populations. This tool showed a reliability coefficient (Cronbach’s α) of 0.83, confirmed also in the Italian validation (α = 0.835; [41], indicating high internal consistency and, thus, is widely used in both clinical and research settings.

The Revised Fibromyalgia Impact Questionnaire (FIQ-R) is a comprehensive tool used to assess the impact of FM on a patient’s health status across three domains: function, overall impact, and symptoms [42]. The 21 questions are based on an 11-point rating scale (0–10) and refer to the past 7 days. The domain “Function” consists of nine items that assess the patient’s ability to perform daily activities; the “Overall impact” domain includes two items assessing the overall impact of FM on work or daily activities, and the domain “Symptoms” consists of ten items that evaluate the severity of symptoms commonly associated with FM, such as pain, fatigue, sleep disturbance, stiffness, anxiety, and depression. The total FIQ-R score ranges from 0 to 100, with higher scores indicating greater impairment and symptom severity. The FIQ-R shows excellent internal consistency (Cronbach alpha = 0.95), confirmed also in its Italian version (α = 0.94) [43].

#### 2.2.2. Psychopathology of Interest (Anxiety and Depression)

The Beck Depression Inventory-II (BDI-II) is a widely used self-report tool for assessing the severity of depressive symptoms in adolescents and adults [44]. It consists of 21 multiple-choice questions covering symptoms experienced in the past two weeks. These symptoms are categorized into various domains, encompassing somatic-affective manifestations to cognitive manifestations. The total score, from 0 to 63, reflects severity of depressive symptoms: 0–13 indicates minimal depression, 14–18 mild, 19–29 moderate, and 30–60 severe. The Italian validated version demonstrated strong internal consistency, with a Cronbach’s α coefficient of 0.840 [45].

The Generalized Anxiety Disorder 7-Item (GAD-7) is a self-report tool developed by Spitzer in 2006 to assess anxiety symptoms over the past two weeks [46]. Comprising seven multiple-choice items, each item is scored on a scale from 0 to 3. The seven items are scored from 0 to 3, with total scores indicating anxiety severity: 0–4 minimal, 5–9 mild, 10–14 moderate, and 15 or higher indicating severe anxiety. The Italian version of the scale [47] has demonstrated excellent internal consistency and good stability (α = 0.89 and composite reliability index = 0.90).

#### 2.2.3. Stress and Traumatic Experiences

The Perceived Stress Scale (PSS-10), developed by Cohen et al. in 1983, is a 10-item self-report tool assessing individuals’ perceptions of stress over the past month [48]. Participants rate their experiences on a Likert scale from 0 (never) to 4 (very often), with total scores ranging from 0 to 40. Although definitive cutoff points for stress levels are not established, higher scores typically indicate greater perceived stress. The PSS is widely validated and reliable across diverse populations [49,50].

The Life Stressor Checklist-Revised (LSC-R) is a self-report tool developed by Wolfe in 1996 to assess an individual’s exposure to 30 traumatic or stressful life events, such as accidents, assaults, and bereavements. Respondents answer “yes” or “no” to indicate whether they have experienced each event, providing both the perceived impact and the individual reaction. The LSC-R is commonly used to measure “trauma load” with three common methods of scoring. Score 1 counts the total number of stressors experienced (0–30), Score 2 calculates a cumulative impact score (0–150) based on the perceived impact of events, and Score 3 captures events that meet the DSM-IV Posttraumatic Stress Disorder (PTSD) Criteria A [51].

### 2.3. Statistical Analysis

All computations were performed using RStudio for MAC OS (version 2024.04.2+764). The relationships between the FM components (i.e., pain, fatigue, sleep), psychopathology area of interest (i.e., anxiety and depression) and stress-trauma dimension (i.e., lifetime trauma load and perceived stress) were evaluated by a nonparametric correlation matrix based on Kendall’s tau-B coefficients. A correlogram was then drawn using a Šidák corrected threshold for the significance level.

The relationship between pain and depression was further evaluated by Structural Equation Modeling (SEM) path analysis (i.e., Maximum likelihood with robust standard errors—MLR method). In the model SEM0, we estimated the loadings of the three indicators (i.e., BPI-S, BPI-I, and PCS total score) of the latent variable pain. In the model SEM1, we evaluated the direct and indirect effects of pain on depression level (BDI-II total score), including fatigue severity (FAS total score), sleep quality (PSQI total score), and perceived stress level (PSS total score) as mediators. In the model SEM2, we built the inverse model to estimate the direct and indirect effects of depression on pain with the same mediators. Then, both SEM1 and SEM2 were included in a combined model, the model SEM3, to evaluate the bidirectional/reciprocal relationship between pain and depression.

The Robust Comparative Fit Index (CFI), Robust Tucker–Lewis Index (TLI), and Robust Root Mean Square Error of Approximation (RMSEA) were calculated to assess the fitting properties of models. Moreover, post hoc power analyses based on the noncentral χ^2^ distribution were conducted, assuming a population RMSEA of 0.05. Power calculations used the effective sample size and model-specific degrees of freedom extracted from the fitted SEM models.

Eventually, a network analysis was conducted to explore the relationship between FM components (i.e., pain as BPI-S, BPI-I, and PCS total score; fatigue as FAS total score; sleep quality as PSQI total score; and impact on daily life as FIQ-R total score), psychopathology (depression as BDI-II total score and anxiety as GAD total score), and stress trauma (perceived stress as PSS and life events as LSC-R scores). The method used is the Gaussian graphical model using Graphical least absolute shrinkage and selection operator based on the Extended Bayesian Information Criterion method (EBICGlasso), with a tuning parameter (*γ*) of 0.25 and 1000 nonparametric bootstrap subsets.

## 3. Results

The present study included a sample of 50 patients with diagnosis of FM, predominantly female (96%), with a mean age of 50.4 years (SD = 9.9; see Supplementary Material Table S1 for further details). Notably, more than half of the sample had comorbid chronic pain syndrome (62%) or history of other chronic syndromes (52%). The clinical features of the sample revealed significant issues related to pain, mental health, stress, fatigue, sleep quality, and the overall impact of FM on daily life (Table S1). The sample exhibited moderate to severe levels of pain severity, pain interference, depression and anxiety, a high tendency to catastrophize, moderate level of perceived stress and fatigue, overall poor sleep quality and a substantial impact of FM on daily life.

## 4. Relationship Between Pain and Depression

### 4.1. Bivariate Analysis

**Figure 1:**
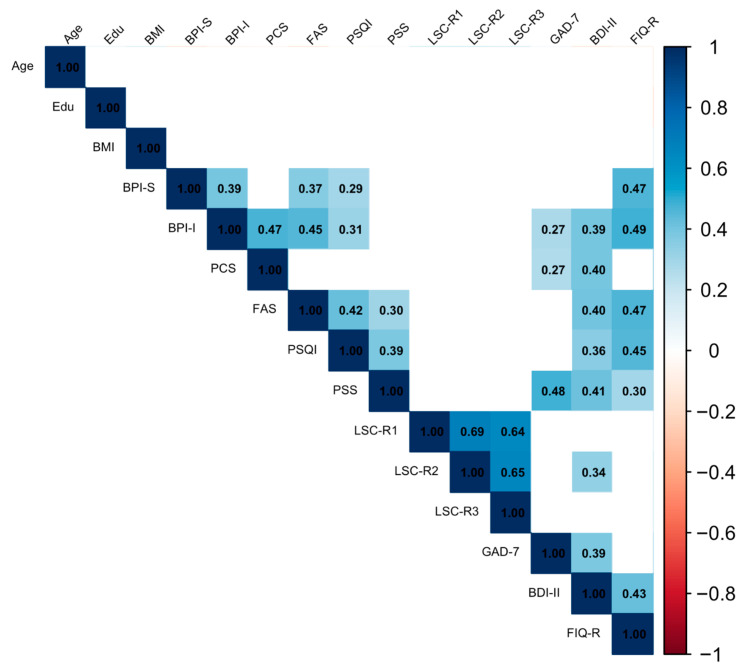
Correlogram including relevant psychopathological and fibromyalgia clinical variables. Note: BMI = Body Mass Index; BPI-S = Brief Pain Inventory Severity; BPI-I = Brief Pain Inventory Interference; PCS = Pain Catastrophizing Scale; PSQI = Pittsburgh Sleep Quality Index; PSS = Perceived Stress Scale-10; LSC-R1 = Life Stressor Checklist score 1; LSC-R2 = Life Stressor Checklist score 2; LSC-R3 = Life Stressor Checklist score 3; GAD-7 = Generalized Anxiety Disorder-7; BDI-II = Beck Depression Inventory-II; FIQ-R = Revised Fibromyalgia Impact Questionnaire.

According to the nonparametric correlation matrix (Figure 1 and Supplementary Material Table S2), severity of depressive symptoms (BDI-II total score) positively correlated with almost all the observed clinical variables of FM, including pain interference (BPI-I, tau-B = 0.39, *p* = 0.001), pain catastrophizing (PCS, tau-B = 0.40, *p* < 0.001), fatigue severity (FAS, tau-B = 0.40, *p* < 0.001), sleep quality (PSQI, tau-B = 0.36, *p* = 0.002), and FM impact on daily life (FIQ-R, tau-B = 0.43, *p* < 0.001) as well as perceived stress (PSS, tau-B = 0.41, *p* < 0.001), anxiety (GAD, tau-B = 0.39, *p* < 0.001), and the individual’s subjective rating of the event’s impact over the past year (LSC2, tau-B = 0.34, *p* = 0.001). Notably, depression did not show a significant correlation with pain severity (BPI-S, tau-B = 0.15, *p* = 0.107).

Regarding pain, higher pain interference (BPI-I) and pain severity (BPI-S) were linked to poorer sleep quality (PSQI, tau-B = 0.31, *p* < 0.001 and tau-B = 0.29, *p* = 0.002, respectively), greater impact of FM on daily life (FIQ-R, tau-B = 0.49, *p* < 0.001 and tau-B = 0.47, *p* < 0.001, respectively), and increased fatigue (FAS, tau-B = 0.45, *p* < 0.001 and tau-B = 0.37, *p* < 0.001, respectively).

### 4.2. Path Analysis

The model SEM0 showed that the pain latent variable was well explained by all three indicators (χ^2^(3) = 46.8, *p* < 0.001; standardized estimates of loading: BPI-S = 1.057, BPI-I = 0.618, PCS total score = 0.551). The detailed results for all the models are presented in Table 1 (see Supplementary Material Figures S1–S3 for the path diagrams).

Model SEM1 showed the following fit indices: χ^2^(11) = 24.7, *p* < 0.010; CFI = 0.89; TLI = 0.78; RMSEA = 0.17. In this model, pain severity did not affect depression level either directly or indirectly (i.e., mediated by fatigue severity and by perceived stress level), but it exerted a significant effect on fatigue severity and sleep quality. Perceived stress level is the only significant predictor of depression severity.

Model SEM2 yielded fit indices of χ^2^(11) = 28.0, *p* = 0.003; CFI = 0.87; TLI = 0.75; RMSEA = 0.18. In contrast to SEM1, it revealed that depression level had a direct effect on pain, being a significant predictor of pain together with fatigue severity. Moreover, depression level exerted a significant effect on fatigue severity, sleep quality, and perceived stress. Therefore, depression had an indirect effect on pain mediated by fatigue (Table 1).

The combined model (SEM3) showed fit indices of χ^2^(4) = 13.1, *p* = 0.011; CFI = 0.96; TLI = 0.77; RMSEA = 0.17. Although it revealed no significant direct or indirect relationships between depression and pain, it did highlight significant pathways between depression and fatigue. Specifically, depression level significantly predicted fatigue severity, which in turn significantly predicted pain. Although pain significantly predicted sleep quality, this pathway did not ultimately contribute to depression level. Finally, perceived stress level was found to be a significant predictor of depression level (see Table 1 for full model results). Detailed results of these post hoc power analyses are reported in Supplementary Material Table S3.

### 4.3. Network Analysis

**Figure 2:**
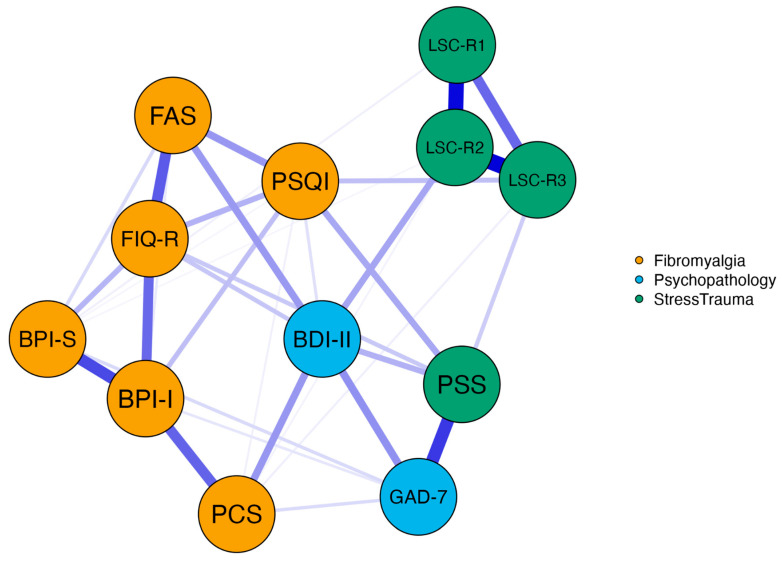
Network analysis of fibromyalgia components, psychopathology, and stress-trauma dimension. Note: The blue lines indicate that all edge weights are positive. Stronger regularized partial correlations are represented by darker and thicker edges. Node colors indicate community. BPI-S = Brief Pain Inventory Severity; BPI-I = Brief Pain Inventory Interference; PCS = Pain Catastrophizing Scale; FAS = Fatigue Assessment Scale; PSQI = Pittsburgh Sleep Quality Index; PSS = Perceived Stress Scale-10; LSC-R1 = Life Stressor Checklist score 1; LSC-R2 = Life Stressor Checklist score 2; LSC-R3 = Life Stressor Checklist score 3; GAD-7 = Generalized Anxiety Disorder-7; BDI-II = Beck Depression Inventory-II; FIQ-R = Revised Fibromyalgia Impact Questionnaire.

The network included 12 variables (nodes) representing FM components, psychopathology, and stress trauma (Figure 2), resulting in 34 non-zero edges (out of a possible 66) and indicating a moderate level of connectivity (sparsity = 0.485). Edge weights revealed strong connections within both the FM and trauma-related variable clusters, while psychopathology variables were less interconnected and showed stronger ties to stress than to trauma.

Centrality analysis highlighted depression as a key node, exhibiting the highest betweenness (2.309) and closeness (2.253) centrality. This suggests that depression plays a crucial role in bridging different symptom clusters and maintaining close relationships with other symptoms. Specifically, depression is the only clinical dimension linked to both FM components and trauma load. Furthermore, pain interference (BPI-I) exhibited high strength centrality (1.238), underscoring its strong influence in the network (see also Supplementary Material Tables S4, S5 and Figure S4).

## 5. Discussion

Fibromyalgia (FM), within the broader epidemiological category of chronic pain conditions, represents a relevant public health burden because of its association with disability, reduced quality of life, productivity loss, and substantial healthcare utilization and societal costs [4,5,6,7]. In this context, comorbid depressive symptoms are not only clinically relevant but also potentially important from a health-system perspective, as depression in chronic pain populations is associated with worse functional outcomes and higher healthcare costs, thereby amplifying burden beyond symptom severity alone [12,13,14]. Against this public health background, the present study contributes a clinically oriented contribution: depressive symptoms appear central within the FM symptom structure, and their position in the network/SEM may help explain why depression is repeatedly linked to disability and service needs at the population level.

Through a comprehensive analysis of psychological factors and FM-related components, the present study highlights the critical role of depression in fibromyalgia. Our findings confirm that depressive symptoms are strongly associated with all clinical components of FM, including sleep disturbances, fatigue, perceived stress levels, and impact on daily life. A substantial body of literature emphasizes that FM and depression share a significant overlap in pathophysiology, with several mechanisms having been proposed [52,53,54].

Central sensitization, a mechanism of heightened pain sensitivity, has been extensively studied in both FM and depression, revealing shared neurobiological bases, including altered gene expression and neuroplasticity [55,56,57,58,59]. In addition, genetic and epigenetic factors involving key neurotransmitter systems have been implicated in vulnerability to both conditions [60,61], including polymorphisms of the serotonin transporter gene (5-HTT) commonly studied in MDD [62]. Exposure to stressors and traumatic experiences may further contribute by activating shared inflammatory and neuroendocrine pathways, with converging evidence of neuroimmune alterations such as microglial activation in both FM and depression [63,64,65,66,67]. Together, these converging mechanisms provide a plausible biological background for the strong associations observed between depressive symptoms and FM-related clinical dimensions.

Within this context, previous studies have variably conceptualized the FM–depression relationship as bidirectional or as part of a broader affective spectrum [10,68,69]. Consistent with this literature, the present study identified a significant association between depression and pain, with depression emerging as a central and strongly connected dimension within the SEM and network frameworks.

Beyond confirming the centrality of depressive symptoms within the FM symptom profile, the present study extends existing work by clarifying how depression and pain are interconnected within a broader clinical structure. Specifically, by testing alternative model-based directions of association, our findings indicated that an inverse pain–depression model, whereby depressive symptoms were associated with pain primarily through fatigue, was more consistent with the observed data than models assuming a direct or reciprocal association. Network analysis further supported this pattern by highlighting depressive symptoms as a central node linking multiple FM-related dimensions.

Among the FM-related dimensions examined, fatigue emerged as a particularly relevant intervening factor in the association between depressive symptoms and pain. Fatigue, defined as overwhelming physical and mental exhaustion, is highly prevalent in several chronic pain conditions, including FM, indicating a potential etiological link [70]. Inflammation, autonomic dysfunction and central sensitization have been identified as relevant pathophysiological mechanisms in the development of fatigue [71]. Moreover, fatigue seems to share neurobiological underpinnings with psychiatric conditions like depression [72,73], as many studies showed that both pain and depression impact fatigue, which in turn affects physical functioning [74,75]. This overlap reinforces the notion that fatigue is not merely a byproduct of pain and depression but a core component of FM dysfunction.

Some authors have proposed the mediating role of fatigue in the relationship between pain and depression [76]. While this view is somewhat consistent with the findings of the present study, a key distinction emerges: no evidence was found to support the mediation of fatigue on the effect of pain on depression. Instead, we took an additional step by testing the inverse relationship, where fatigue significantly mediates the relationship between depression and pain.

The present study emphasizes the prominent role of depression in the clinical profile of FM, as supported by the network analysis, acting as a bridge between trauma/stress and key FM symptoms, including pain, fatigue, sleep quality, and impact on daily life. Depression emerges not only as a central and clinically relevant dimension within the FM symptom profile but is also closely associated with both emotional and physical dysfunction. This aligns with literature that highlights the predominant negative effect tone in FM patients and the efficacy of antidepressant treatments [18,19,77].

Although some authors have proposed stress as an etiopathogenetic factor in emotional regulation imbalance, contributing to heightened pain sensitivity and emotional distress in FM patients [22,73,77], in the present study, perceived stress does not appear to play a central role in the FM symptom network. However, the SEM model does indicate that stress correlates with depressive symptoms, which in turn influence fatigue, pain, and sleep quality. This suggests a multifaceted interplay between psychological and physical symptoms rather than a straightforward linear progression, with stress contributing to, but not necessarily driving, the overall symptom burden in FM.

However, the simplistic notion of a linear cause-and-effect relationship is insufficient. Instead, the evidence points to complex, dynamic interactions among these various manifestations and mechanisms, which influence each other before, during, and after FM onset [77,78,79].

Taken together, these clinical findings also have implications beyond individual-level symptom management. From a public health and health services standpoint, the present findings also provide a rationale for systematic detection of depressive symptoms within FM/chronic pain care pathways. Population-based evidence shows that comorbid anxiety/depression in chronic pain is associated with substantial work limitation and functional impact [13,14], and clinical service data indicate higher healthcare costs among chronic pain patients with depression [12]. Accordingly, depressive symptom assessment should be considered not only a clinical priority but also a potential leverage point for reducing disability and healthcare demand [80,81].

In addition, the broader literature increasingly emphasizes integrated and multidisciplinary approaches for chronic pain management, rather than siloed single-modality care, particularly when mental health comorbidity is present [81,82]. Within this framework, identifying depressive symptoms as a central node in the FM clinical structure may help inform care pathway planning within a broader policy framework that increasingly recognizes chronic pain as a disease entity and emphasizes epidemiological evidence to guide resource allocation [83,84].

These findings underscore the importance of systematically assessing depressive symptoms in patients with fibromyalgia, given their central position within the symptom network and their significant associations with pain and fatigue. Although causal inferences cannot be drawn from this cross-sectional design, the observed pattern is consistent with multidimensional models of FM emphasizing the interplay between affective processes and symptom burden [77,78]. In line with this interpretation, integrated strategies addressing mood symptoms, fatigue, and pain-related dysfunction may be clinically appropriate [17,18,19]. Moreover, embedding mental health screening and referral/stepped interventions into routine FM/chronic pain services may have health-system relevance by improving identification of high-need patients and potentially mitigating downstream disability and service utilization [80,81].

## 6. Limitations

The study has several limitations. First, its cross-sectional design precludes the ability to infer causality between depression, pain, fatigue, and other FM-related symptoms. Longitudinal studies are needed to confirm the temporal relationships and causal pathways suggested by the SEM model. Second, the sample size may limit the generalizability of the findings and substantially constrain statistical power. Post hoc power estimates based on the global χ^2^ test indicate that the SEM models were adequately powered only to detect deviations from close fit of at least medium magnitude. Importantly, χ^2^-based power reflects global model sensitivity and does not directly index the power to detect individual structural paths. In combination with model complexity, the small sample size may also have contributed to less-than-ideal global fit indices in some SEMs, indicating that these analyses should be considered exploratory. Accordingly, non-significant pathways (particularly in the SEM3 model) should be interpreted as inconclusive rather than as evidence of the absence of an effect, and smaller pathway effects cannot be ruled out. Third, the sample was predominantly female, which reflects the epidemiology of fibromyalgia but limits the generalizability of the results to male patients and precludes sex-specific analyses. Finally, although the study identified key factors such as fatigue and perceived stress, the complexity of FM requires exploring additional aspects, such as the retrospective collection of clinical data related to depression or pain, which could offer valuable insights, given the dynamic and fluctuating nature of these symptoms in FM.

## 7. Conclusions

The findings of this study highlight the complex relationship between depression and pain in fibromyalgia (FM), emphasizing the central role of depression within the symptom profile and its close association with both emotional and physical dysfunction. Fatigue emerged as a significant mediator between depression and pain, while the role of perceived stress, although not central, contributes to the overall symptom burden in FM. These interactions underscore the multifaceted nature of FM, where psychological and physical symptoms are intricately interconnected through shared mechanisms.

These results support the clinical relevance of systematically assessing depressive symptoms in FM, as mood-related processes appear closely linked to pain, fatigue, sleep disturbance, and functional impact. At the same time, the identification of depression as a structurally prominent dimension may also have implications for organizing care pathways and health-service planning. Integrating mental health evaluation and coordinated interventions within chronic pain management models may, therefore, represent not only a clinically appropriate strategy but also a relevant approach from a public health perspective, particularly in conditions such as FM that contribute substantially to disability and service demand.

Longitudinal studies are warranted to determine whether targeting central symptom dimensions translates into measurable improvements in both individual outcomes and health-system burden.

## Figures and Tables

**Table 1:** Path analysis of the relationship between depression and pain with the mediating effect of fatigue, sleep quality and stress.

	Estimate (B)	95% CI		β	*p*
		LL	UL		
Model 1. Path from pain to depression					
Pain → BDI-II (c)	2.149	−1.136	5.433	0.332	0.200
FAS → BDI-II (b1)	0.384	−0.152	0.921	0.237	0.160
PSQI → BDI-II (b2)	−0.341	−1.315	0.633	−0.096	0.492
PSS → BDI-II (b3)	0.607	0.224	0.989	0.371	0.002 *
Pain → FAS (a1)	2.719	0.882	4.555	0.681	0.004 *
Pain → PSQI (a2)	1.215	0.532	1.897	0.667	<0.001 *
Pain → PSS (a3)	1.784	−0.362	3.930	0.451	0.103
Pain → FAS → BDI-II (a1 ∗ b1)	1.045	−0.344	2.434	0.161	0.140
Pain → PSQI → BDI-II (a2 ∗ b2)	−0.414	−1.662	0.834	−0.064	0.515
Pain → PSS → BDI-II (a3 ∗ b3)	1.082	−0.145	2.309	0.167	0.084
Model 2. Path from depression to pain (inverse model)					
BDI-II → Pain (c)	0.044	0.005	0.082	0.261	0.025 *
FAS → Pain(b1)	0.084	0.017	0.151	0.309	0.014 *
PSQI → Pain (b2)	0.200	−0.044	0.444	0.334	0.108
PSS → Pain (b3)	−0.030	−0.134	0.074	−0.110	0.569
BDI-II → FAS (a1)	0.347	0.205	0.489	0.564	<0.001 *
BDI-II → PSQI (a2)	0.122	0.053	0.190	0.434	<0.001 *
BDI-II → PSS (a3)	0.350	0.194	0.505	0.573	<0.001 *
BDI-II → FAS → Pain (a1 ∗ b1)	0.029	0.001	0.057	0.174	0.040 *
BDI-II → PSQI → Pain (a2 ∗ b2)	0.024	−0.010	0.059	0.145	0.164
BDI-II → PSS → Pain (a3 ∗ b3)	−0.011	−0.046	0.025	−0.063	0.556
Model 3. Path between pain and depression (combined model)					
Pain → BDI-II (c)	0.783	−3.598	5.164	0.134	0.726
FAS → BDI-II (b1)	0.043	−1.005	1.091	0.026	0.936
PSQI → BDI-II (b2)	−2.679	−5.988	0.630	−0.752	0.113
PSS → BDI-II (b3)	1.595	0.014	3.177	0.973	0.048 *
Pain → FAS (a1)	0.079	−1.828	1.986	0.022	0.935
Pain → PSQI (a2)	1.986	0.064	3.908	1.215	0.043 *
Pain → PSS (a3)	0.116	−1.592	1.824	0.033	0.894
BDI-II → Pain (cc)	0.149	−0.055	0.353	0.865	0.154
FAS → Pain (bb1)	0.263	0.048	0.478	0.943	0.016 *
PSQI → Pain (bb2)	−0.575	−1.231	0.080	−0.941	0.085
PSS → Pain (bb3)	−0.057	−0.426	0.312	−0.203	0.761
BDI-II → FAS (aa1)	0.414	0.106	0.723	0.673	0.008 *
BDI-II → PSQI (aa2)	0.030	−0.164	0.224	0.106	0.765
BDI-II → PSS (aa3)	−0.100	−0.580	0.380	−0.164	0.683
Pain → FAS → BDI-II (a1 ∗ b1)	0.003	−0.121	0.127	0.001	0.957
Pain → PSQI → BDI-II (a2 ∗ b2)	−5.320	−14.453	3.814	−0.913	0.254
Pain → PSS → BDI-II (a3 ∗ b3)	0.184	−2.588	2.957	0.032	0.896
BDI-II → FAS → Pain (aa1 ∗ bb1)	0.109	−0.038	0.256	0.635	0.146
BDI-II → PSQI → Pain (aa2 ∗ bb2)	−0.017	−0.120	0.086	−0.099	0.745
BDI-II → PSS → Pain (aa3 ∗ bb3)	0.006	−0.057	0.069	0.033	0.859

Note: β = Standardized estimate; CI = confidence interval; LL = lower limit; UL = upper limit; FAS = Fatigue Assessment Scale; PSQI = Pittsburgh Sleep Quality Index; PSS = Perceived Stress Scale-10; BDI-II = Beck Depression Inventory-II. * *p* < 0.05.

## Data Availability

The data that support the findings of this study are available from the corresponding author upon reasonable request.

## Supplementary Materials

The following supporting information can be downloaded at https://www.mdpi.com/article/10.3390/ijerph23030316/s1. Table S1. Sociodemographic and clinical characteristics of the sample (N=50); Table S2. *p*-value from the Kendall's tau correlation analysis between depression, pain-related measures, and other clini-cal variables in fibromyalgia patients; Table S3. Post-hoc power analyses for SEM models; Table S4. Centrality indices measures of network analysis; Table S5. Matrix of edge weights of network analysis; Figure S1. Structural Equation Model: mediation effect of fatigue, stress and sleep disturbance in the effect of pain to depression (SEM 1); Figure S2. Structural Equation Model: mediation effect of fatigue, stress and sleep disturbance in the effect of depression to pain (SEM 2); Figure S3. Structural Equation Model: mediation effect of fatigue, stress and sleep disturbance in the reciprocal effect between pain and depression (SEM 3); Figure S4. Centrality indices plot of network analysis.
