# Supplementary material for: The Relationship Between Pain and Depression in Fibromyalgia: Structural Equation Modeling and Network Analysis
Source: Int J Environ Res Public Health. 2026 Mar 4;23(3):316. doi: 10.3390/ijerph23030316 (PMC13026093; doi:10.3390/ijerph23030316)
Supplement: Supplementary file 1 [file ijerph-23-00316-s001.zip › ijerph-4095362-supplementary.pdf]

SUPPLEMENTARY MATERIAL

Table S1. Sociodemographic and clinical characteristics of the sample (N=50).

	**n (%)**
Female	48 (96.0)
Education level	
Lower secondary school	19 (38.0)
Upper secondary school	22 (44.0)
Bachelor's degree or more	9 (18.0)
Employment	
Employed	33 (66.0)
Unemployed	14 (28.0)
Retired	3 (6.0)
Marital status	
Single	4 (8.0)
Common-law couple	4 (8.0)
Married	27 (54.0)
Separated/divorced	15 (30.0)
Living condition	
Living alone	4 (8.0)
Living with family	46 (92.0)
Diet regimen	
No diet restriction	33 (66.0)
Restricted regimen	10 (20.0)
Allergy/intolerance	7 (14.0)
Smoke habits, yes	17 (34.0)
Alcohol consumption, yes	10 (20.0)
Chronic pain condition, yes	31 (62.0)
Other chronic condition, yes	26 (52.0)
Depressive disorder, yes	11 (22.0)
Anxiety disorder, yes	6 (12.0)
Eating disorder, yes	2 (4.0)
Substance use disorder, yes	1 (2.0)
	M (SD)
Age (years)	50.4 (9.9)
BMI (kg/m^2)	24.9 (7.0)
BPI-S	6.9 (1.9)
BPI-I	7.5 (2.1)
PCS total	31.4 (11.4)
FAS total score	35.3 (7.1)
PSS-10 total score	24.9 (7.1)
GAD-7 total score	14.2 (4.9)
BDI-II total score	24.1 (11.6)
FIQ-R total score	73.3 (16.2)
LSC-R	
LSC-R score 1	7.0 (4.0)
LSC-R score 2	18.5 (14.6)
LSC-R score 3	3.0 (2.9)

Note: BMI = Body Mass Index; BPI-S = Brief Pain Inventory Severity; BPI-S = Brief Pain Inventory Interference (BPI-I); PCS = Pain Catastrophizing Scale; FAS = Fatigue Assessment Scale; PSS-10 = Perceived Stress Scale-10; GAD-7 = Generalized Anxiety Disorder-7; BDI-II = Beck Depression Inventory-II; FIQ-R = Revised Fibromyalgia Impact Questionnaire; PSQI = Pittsburgh Sleep Quality Index; LSC-R = Life Stressor Checklist - Revised.

Table S2. *P-value* from the Kendall's tau correlation analysis between depression, pain-related measures, and other clinical variables in fibromyalgia patients.

	Age	Edu	BMI	BPI-S	BPI-I	PCS	FAS	PSQI	PSS	LSC-R1	LSC-R2	LSC-R3	GAD-7	BDI-II	FIQ-R
**Age**	-														
**Education years**	0.761	-													
**BMI**	0.247	0.406	-												
**BPI-S**	0.385	0.005	0.711	-											
**BPI-I**	0.389	0.117	0.786	0.000	-										
**PCS**	0.613	0.766	0.714	0.015	0.000	-									
**FAS**	0.173	0.247	0.466	0.000	0.000	0.027	-								
**PSQI**	0.921	0.156	0.137	0.002	0.000	0.009	0.000	-							
**PSS**	0.744	0.600	0.245	0.201	0.038	0.011	0.002	0.000	-						
**LSC-R1**	0.099	0.184	0.282	0.529	0.733	0.213	0.957	0.693	0.074	-					
**LSC-R2**	0.098	0.098	0.240	0.720	0.528	0.036	0.773	0.621	0.037	0.000	-				
**LSC-R3**	0.314	0.048	0.416	0.639	0.868	0.067	0.998	0.309	0.024	0.000	0.000	-			
**GAD-7**	0.973	0.156	0.817	0.006	0.001	0.002	0.019	0.009	0.000	0.509	0.221	0.414	-		
**BDI-II**	0.095	0.459	0.621	0.107	0.001	0.000	0.000	0.002	0.000	0.043	0.001	0.015	0.000	-	
**FIQ-R**	0.319	0.392	0.462	0.000	0.000	0.006	0.000	0.000	0.000	0.548	0.418	0.502	0.012	0.000	-

Edu = Education years; BMI = Body Mass Index; BPI-S = Brief Pain Inventory Severity; BPI-I = Brief Pain Inventory Interference; PCS = Pain Catastrophizing Scale; FAS = Fatigue Assessment Scale; PSQI = Pittsburgh Sleep Quality Index; PSS = Perceived Stress Scale-10; LSC-R1 = Life Stressor Checklist score 1; LSC-R2 = Life Stressor Checklist score 2; LSC-R3 = Life Stressor Checklist score 3; GAD-7 = Generalized Anxiety Disorder-7; BDI-II= Beck Depression Inventory; FIQ-R = Revised Fibromyalgia Impact Questionnaire.

Table S3. Post-hoc power analyses for SEM models.

Model	N	df	RMSEA	Alpha	Power	NCP
SEM1	50	11	0.05	0.05	0.092	1.348
SEM2	50	11	0.05	0.05	0.092	1.348
SEM3	50	4	0.05	0.05	0.076	0.490

NCP = Non-Centrality Parameter; RMSEA = Root Mean Square Error of Approximation, SEM = Structural Equation Model.

Table S4. Centrality indices measures of network analysis.

Variable	Betweenness	Closeness	Strength	Expected influence
BPI-S	-0.981	-1.439	-1.220	-1.220
BPI-I	1.097	0.043	1.238	1.238
PCS	0.231	0.378	-1.486	-1.486
FAS	-0.289	0.665	-0.517	-0.517
PSQI	-0.289	0.743	-0.239	-0.239
PSS	-0.635	-0.350	-0.119	-0.119
LSC-R1	-0.981	-1.300	-0.818	-0.818
LSC-R2	0.924	-0.235	1.294	1.294
LSC-R3	-0.289	-0.820	0.604	0.604
GAD-7	-0.981	-0.263	-0.788	-0.788
BDI-II	2.309	2.253	1.021	1.021
FIQ-R	-0.115	0.325	1.029	1.029

BPI-S = Brief Pain Inventory Severity; BPI-I = Brief Pain Inventory Interference; PCS = Pain Catastrophizing Scale; FAS = Fatigue Assessment Scale; PSQI = Pittsburgh Sleep Quality Index; PSS = Perceived Stress Scale-10; LSC-R1 = Life Stressor Checklist score 1; LSC-R2 = Life Stressor Checklist score 2; LSC-R3 = Life Stressor Checklist score 3; GAD-7 = Generalized Anxiety Disorder-7; BDI-II= Beck Depression Inventory; FIQ-R = Revised Fibromyalgia Impact Questionnaire.

Table S5. Matrix of edge weights of network analysis.

Variable	BPI-S	BPI-I	PCS	FAS	PSQI	FIQ-R	GAD-7	BDI-II	PSS	LSC-R1	LSC-R2	LSC-R3
BPI-S	0.000	0.338	0.000	0.074	0.019	0.140	0.072	0.000	0.000	0.029	0.018	0.000
BPI-I	0.338	0.000	0.292	0.044	0.123	0.282	0.050	0.000	0.000	0.000	0.000	0.000
PCS	0.000	0.292	0.000	0.000	0.028	0.000	0.071	0.197	0.000	0.000	0.024	0.032
FAS	0.074	0.044	0.000	0.000	0.197	0.308	0.000	0.194	0.000	0.000	0.000	0.000
PSQI	0.019	0.123	0.028	0.197	0.000	0.148	0.000	0.060	0.168	0.000	0.000	0.122
FIQ-R	0.140	0.282	0.000	0.308	0.148	0.000	0.000	0.108	0.104	0.000	0.000	0.000
GAD-7	0.072	0.050	0.071	0.000	0.000	0.000	0.000	0.209	0.367	0.000	0.000	0.000
BDI-II	0.000	0.000	0.197	0.194	0.060	0.108	0.209	0.000	0.150	0.000	0.171	0.000
PSS	0.000	0.000	0.000	0.000	0.168	0.104	0.367	0.150	0.000	0.000	0.000	0.098
LSC-R1	0.029	0.000	0.000	0.000	0.000	0.000	0.000	0.000	0.000	0.000	0.448	0.286
LSC-R2	0.018	0.000	0.024	0.000	0.000	0.000	0.000	0.171	0.000	0.448	0.000	0.477
LSC-R3	0.000	0.000	0.032	0.000	0.122	0.000	0.000	0.000	0.098	0.286	0.477	0.000

BPI-S = Brief Pain Inventory Severity; BPI-I = Brief Pain Inventory Interference; PCS = Pain Catastrophizing Scale; FAS = Fatigue Assessment Scale; PSQI = Pittsburgh Sleep Quality Index; FIQ-R = Revised Fibromyalgia Impact Questionnaire; GAD-7 = Generalized Anxiety Disorder-7; BDI-II= Beck Depression Inventory; PSS = Perceived Stress Scale-10; LSC-R1 = Life Stressor Checklist score 1; LSC-R2 = Life Stressor Checklist score 2; LSC-R3 = Life Stressor Checklist score 3.

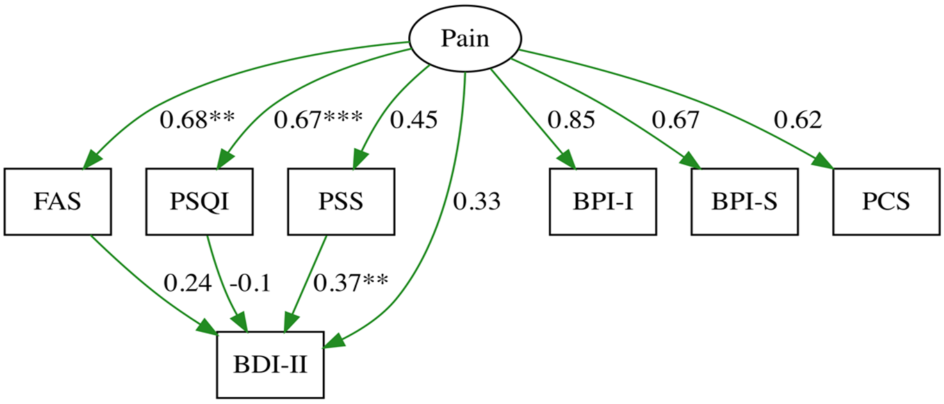

Figure S1. Structural Equation Model: mediation effect of fatigue, stress and sleep disturbance in the effect of pain to depression (SEM 1). BDI-II= Beck Depression Inventory; BPI-I = Brief Pain Inventory Interference; BPI-S = Brief Pain Inventory Severity; FAS = Fatigue Assessment Scale; PCS = Pain Catastrophizing Scale; PSQI = Pittsburgh Sleep Quality Index; PSS = Perceived Stress Scale-10.

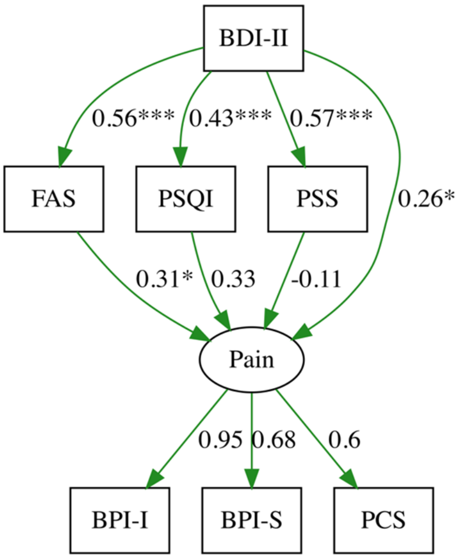

Figure S2. Structural Equation Model: mediation effect of fatigue, stress and sleep disturbance in the effect of depression to pain (SEM 2). BDI-II= Beck Depression Inventory; BPI-I = Brief Pain Inventory Interference; BPI-S = Brief Pain Inventory Severity; FAS = Fatigue Assessment Scale; PCS = Pain Catastrophizing Scale; PSQI = Pittsburgh Sleep Quality Index; PSS = Perceived Stress Scale-10.

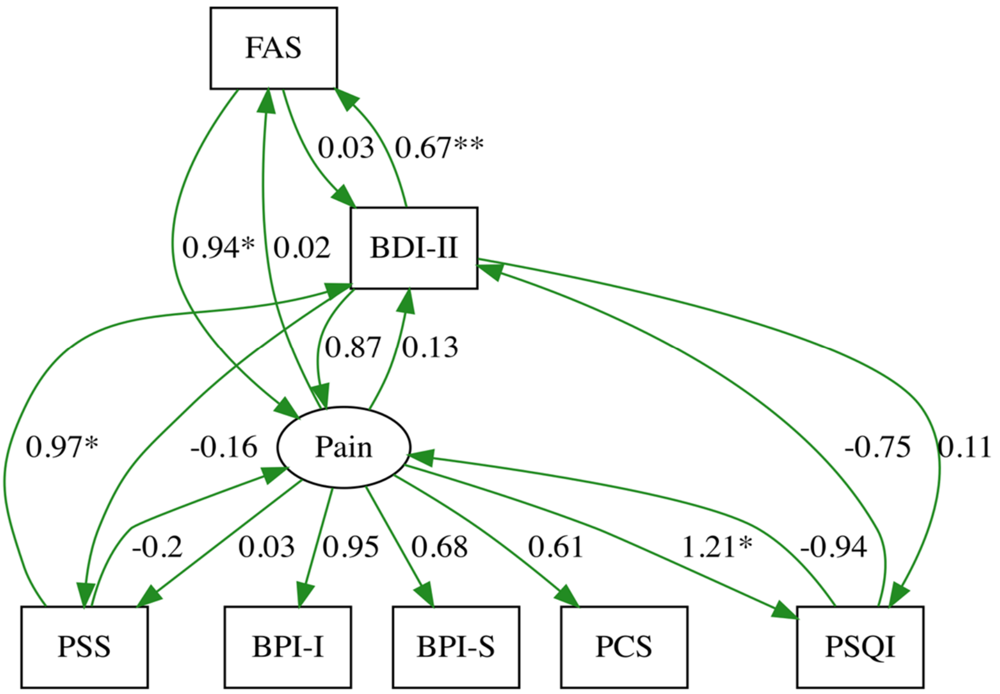

Figure S3. Structural Equation Model: mediation effect of fatigue, stress and sleep disturbance in the reciprocal effect between pain and depression (SEM 3). BDI-II= Beck Depression Inventory; BPI-I = Brief Pain Inventory Interference; BPI-S = Brief Pain Inventory Severity; FAS = Fatigue Assessment Scale; PCS = Pain Catastrophizing Scale; PSQI = Pittsburgh Sleep Quality Index; PSS = Perceived Stress Scale-10.

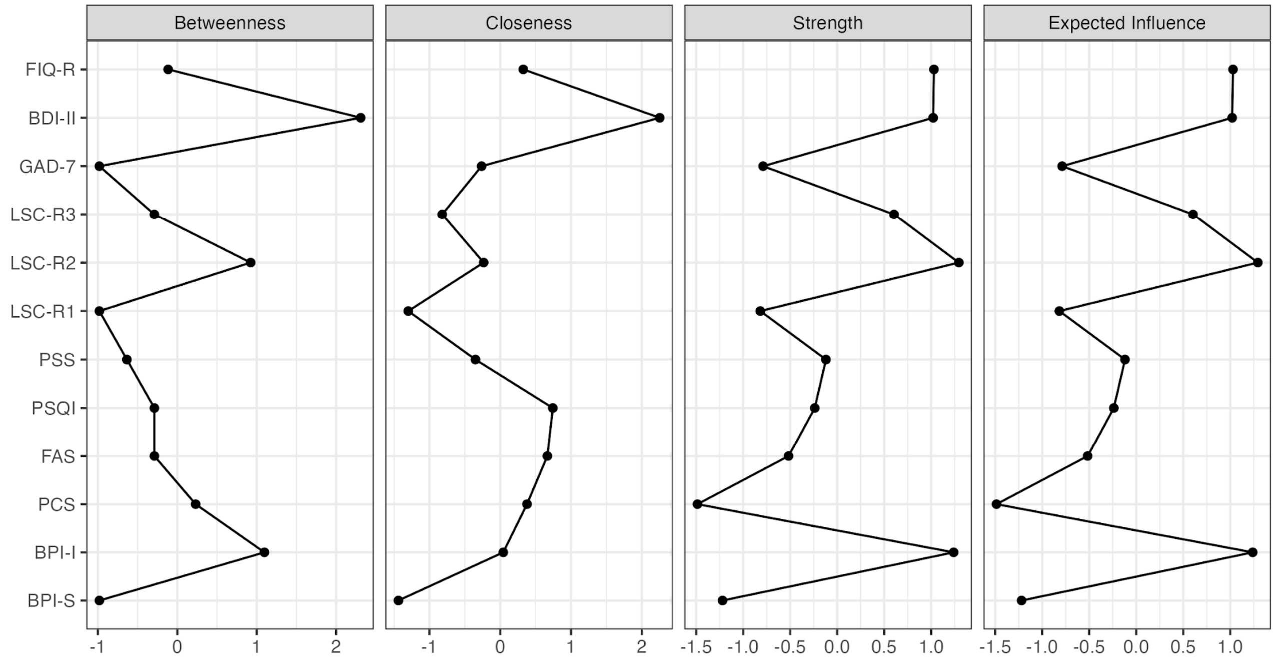

Figure S4. Centrality indices plot of network analysis. BPI-S = Brief Pain Inventory Severity; BPI-I = Brief Pain Inventory Interference; PCS = Pain Catastrophizing Scale; FAS = Fatigue Assessment Scale; PSQI = Pittsburgh Sleep Quality Index; PSS = Perceived Stress Scale-10; LSC-R1 = Life Stressor Checklist score 1; LSC-R2 = Life Stressor Checklist score 2; LSC-R3 = Life Stressor Checklist score 3; GAD-7 = Generalized Anxiety Disorder-7; BDI-II= Beck Depression Inventory; FIQ-R = Revised Fibromyalgia Impact Questionnaire.
